# Promoting Family Communication for Cascade Cancer Genetic Testing With Relational Agent Role-Play: Quasi-Experimental Study

**DOI:** 10.2196/87623

**Published:** 2026-04-21

**Authors:** Timothy Bickmore, Madison Blain, Meghan Underhill

**Affiliations:** 1 Relational Agents Group, Khoury College of Computer Sciences, Northeastern University, 360 Huntington Ave, Boston, MA, 02115, United States, 1 6173735477; 2 School of Nursing, University of Rochester, Rochester, NY, United States

**Keywords:** virtual agents, cascade genetic testing, cancer genetics, cancer risk, communication skills training, embodied conversational agents

## Abstract

**Background:**

If a patient with cancer is identified as having a pathogenic variant, at-risk relatives are eligible for genetic testing, known as cascade testing. However, in the United States, the patient is responsible for informing their family members, and only about 30% of these family members are ultimately informed and complete testing. There is a need to train patients with cancer to communicate risk information and motivate their family members to obtain genetic testing.

**Objective:**

This study evaluates “GRACE,” an online relational agent that trains patients with cancer to talk to their family about cancer risk, including role-play simulations that enable patients to practice communication skills.

**Methods:**

A quasi-experimental study was conducted with 30 crowd workers with cancer. Primary measures included 5-point pre-post self-reported intent, importance, comfort, and confidence to share genetic test information with family members, as well as knowledge of cancer genetics (KnowGene), satisfaction with (10-item satisfaction measure), and usability of (SUS) the relational agent system.

**Results:**

Likelihood of sharing genetic test information increased significantly pre-post from 4.43 (SD 1.04) to 4.67 (SD .66), Wilcoxon (Z=2.07, *P*=.04). Importance of sharing genetic test information increased significantly pre-post from 4.47 (SD .82) to 4.77 (SD .50), Wilcoxon (Z=2.46, *P*=.01). Comfort sharing genetic test information increased pre-post from 4.33 (SD 0.99) to 4.57 (SD 0.90), Wilcoxon (Z=1.811, *P*=.07). Confidence to share genetic test information increased significantly pre-post from 4.33 (SD 0.994) to 4.63 (SD 0.765), Wilcoxon (Z=2.23, *P*=.03). Knowledge of cancer genetics did not increase significantly (mean 13.27, range 1.911 to 13.7, SD 1.932, paired t_29_=1.245, *P*=.22). Participants gave high scores for usability (SUS score=71%) and satisfaction (6.09 SD 0.96 out of 7.0), significantly greater than neutral, t_29_=13.445, *P*<.001) with the relational agent system.

**Conclusions:**

GRACE provides communication skills training and information better enabling patients with cancer to reach out to their families, and our preliminary study indicates a potential for future impact. While results were generally positive, these findings should be interpreted with caution due to limitations in the population included in the pilot, the quasi-experimental design and small sample size. Future development should focus on larger-scale evaluation and in-depth follow-up of family communication dynamics following the use of GRACE.

## Introduction

In the United States, approximately 2 million people are diagnosed annually with cancer, and 5%‐10% of those cancers are due to a pathogenic variant (100,000 to 2,00,000 individuals) in a gene associated with cancer [1]. Once identified, their at-risk relatives are then recommended for testing. In the United States, the average family size includes 4‐6 first degree relatives. Consequently, on average, 600,000 to 1,200,000 individuals are eligible for cancer genetic testing annually, and this number does not include the extended family and therefore is likely much larger [2]. However, due to multiple barriers [3], only 30% of these individuals are informed of their potential cancer risk [4], depriving them of potentially life-saving measures that could prevent cancer or detect it at an early, curable, stage [5]. Additionally, further relatives become eligible for testing once identified, creating a “cascade” effect that enhances family-centered health [5]. In the United States, the responsibility of this cascade cancer risk communication falls on the individual with cancer (the proband), who is often struggling with their diagnosis and treatment, and lacks the knowledge and communication skills to effectively inform their family.

In a recent meta-analysis aiming to understand interventions to facilitate family communication of genetic testing results, 14 interventions were identified that had been evaluated in a randomized controlled trial [6]. Within those results, 3 studies were web-based. Of these, outcomes were mixed, interventions were found acceptable and demonstrated an effect on improving knowledge, while one trial reported no impact. Additional protocol papers and early-stage reports have been identified that are evaluating digital tools for family communication [7-10]. The majority of completed and tested interventions identified in the meta-analysis required human counselor interactions [11]. Current approaches to promoting cascade genetic testing primarily involves the provision of standardized education materials to family members, along with tools to facilitate the transmission of this information from the patient with cancer. While these approaches reduce some barriers, they fail to address the most important ones regarding training and motivating the patient with cancer to reach out to their family members [11]. This research aimed to address these shortcomings by designing and evaluating an online intervention grounded in motivational interviewing theory [12,13] focused on the patient with cancer, and to leverage a user interface medium that is accessible to patients from all levels of health, reading, and computer literacy.

The purpose of this pilot project was to assess whether a relational agent intervention affects patients’ knowledge, intent, and confidence in discussing cancer genetics test results with their family, motivates family members to test. We hypothesize that individuals who engage in the intervention will have increased knowledge of cancer genetic testing and have an intention to discuss results with family members to motivate family cascade testing and confidence in their ability to do so. Satisfaction and usability of the agent was also assessed.

## Methods

### Study Design

Our study report is guided by the Transparent Reporting of Evaluations with Nonrandomized Designs (TREND) reporting structure for nonrandomized studies [14]. The study was a nonrandomized single arm pre-post study. Study participants conducted a single online study session, involving pretest questionnaires, an interaction with the study intervention, GRACE, and post-test questionnaires.

### Participants and Recruitment

All participants were recruited online through the Prolific web-based research platform [15]. The inclusion criteria were the following: (1) age 18 years or older; (2) reside in the United States; (3) speak and read English; and (4) have a current or prior diagnosis of cancer. Prolific’s system prescreens participants and handles all aspects of recruitment automatically, offering enrollment to eligible participants until the recruitment target is met. A total of 30 participants who met the inclusion criteria completed the study. We used four “attention check” questions in the surveys, as is common practice in web-based studies [16-18] but no participants failed more than one; so all participants who completed the study tasks were retained. Participants engaged with the intervention remotely. The intervention and measure collection was completely automated.

### Ethical Considerations

Ethics approval was received from the Institutional Review Board at Northeastern University (reference 25-04-01). All participants completed an informed consent process, which allowed them to opt out of any survey questions or withdraw from the study at any time. The participants were compensated US $15 after completing the survey. All collected data were deidentified.

### Intervention

**Figure 1.**
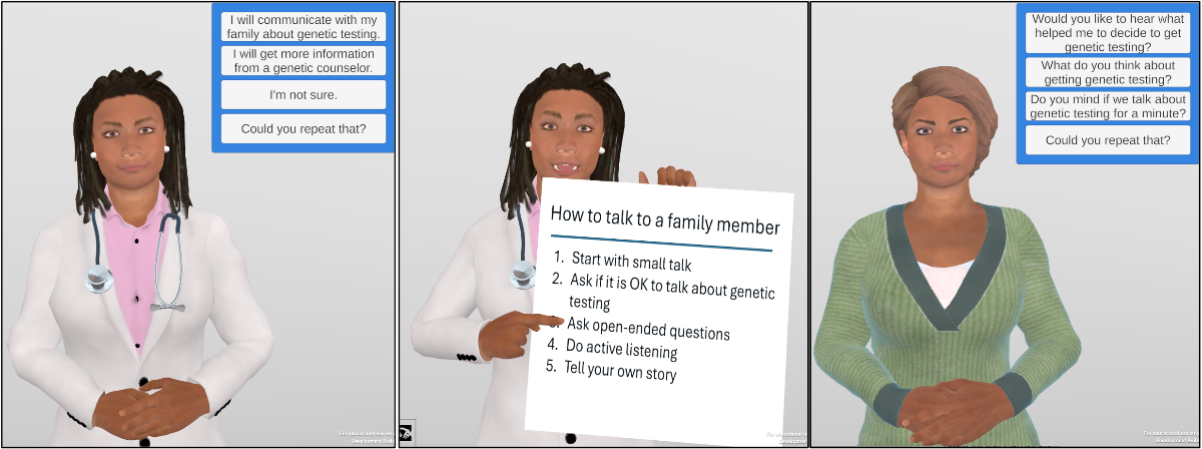
Screen shots of GRACE (Genetic Relational Agent for Cascade), showing: (**L**) the instructional Relational Agent and multiple-choice user input menus; (**M**) giving an overview of family communication; (**R**) the role-play agent.

We developed an online system called “GRACE” (Genetic Relational Agent for Cascade) that features a relational agent (RA) that interacts with patients with cancer using simulated face-to-face conversation (Figure 1). GRACE was deployed as a web-based application developed using the Unity 3D game engine. The RA uses synthetic speech and animation to simulate a face-to-face conversation, and the patient responds with a touch screen or mouse. All dialogue is scripted so that accuracy and safety can be reviewed. This interface has been successfully used in several health interventions with thousands of patients across the health literacy spectrum [19-24], including a pilot study of a RA that provided pretest genetic testing to patients with cancer [25]. The content of GRACE was developed based on previous work within cancer genetics and cancer genetic testing, whose input was provided by health providers, patients, and families as the content was developed. All clinical content was created and approved by experts in cancer genetic counseling and testing. Previous work has verified that the structure, approach, and style of the Relational Agent is acceptable to patients. It is intended that this content take as much or as little time for the patient to review as necessary; however, it is designed to be brief, less than 20 minutes, and can be administered remotely.

The RA first spends several minutes reviewing the basics of cancer genetics, including the concepts of genes, pathogenic variants, inherited risk, what individuals can do to reduce their cancer risk if they know they have a pathogenic variant, and what the genetic testing process involves. It then elicits from the proband the most likely family member they would talk to first about genetic testing. It then covers a series of modules on communication skills, following Buckman’s six-step strategy [16] augmented with techniques from Motivational Interviewing [17], starting with setting the context, building rapport, and asking permission to discuss cancer genetic testing, before progressing to asking open-ended questions, active listening, and relaying their own story about reasons for getting tested and what the process was like for them. For each module, GRACE first provides didactic instruction on the skill, including examples, before allowing the user to practice each skill in a role-play with a different conversational agent that takes on the persona of the family member the proband indicated they would talk to first. At each turn of the role-play, the proband is given the option of choosing from among one correct response and several alternatives representing common communication mistakes. If the user chooses the correct options throughout the role-play, the instructional RA gives positive reinforcement. However, if they make a mistake, the role-play agent provides immediate in situ feedback (eg, acting offended and leaving), the role-play is ended, and the user is given the option of repeating.

### Outcome Measures

Web-based questionnaires were distributed in English to the participants using a Qualtrics web survey.

### Primary Outcome

#### Cancer Genetics Knowledge

Cancer genetics knowledge was assessed before and after the intervention using KnowGene [18], a 19-item instrument designed to assess understanding of general concepts learned within a multigene panel genetic counseling session. A total score is calculated with higher scores indicating higher knowledge.

### Secondary Outcomes

#### Family Communication

Participant attitudes towards sharing genetic test information with their family were assessed before and after the intervention using five five-point scale response items (Table 1). These measures were investigator-initiated based on CSER consortium recommendations [26] and previous trials. Immediately prior to answering each of these questions, participants were prompted with “Assuming you had just been informed that a mutation (pathogenic variant) in one of your genes likely contributed to your cancer.”

**Table 1.** Single-item measures of family communication.

Item	Anchor 1	Anchor 5	Pre mean (SD)	Post mean (SD)	*P* value^a^
How likely are you to share your genetic test results with your immediate family?	Very unlikely	Very likely	4.43 (1.04)	4.67 (0.66)	.04
How important do you believe it is to share your genetic test results with your family members?	Not important	Extremely important	4.47 (0.82)	4.77 (0.50)	.01
How comfortable do you feel about discussing your genetic test results with your family?	Very uncomfortable	Very comfortable	4.33 (0.99)	4.57 (0.90)	.07
How confident do you feel about discussing your genetic test results with your family?	Not confident	Very confident	4.27 (1.02)	4.63 (0.77)	.03

a All *P* values are 2-tailed, Wilcoxon Signed Ranks Tests of pre-post change.

#### Usability and Satisfaction

Usability was assessed after the intervention using the System Usability Scale (SUS) [27]. SUS scores range from 0 to 100, with higher scores indicating greater usability, and scores above 70 indicating good usability [28].

Satisfaction with GRACE was assessed after the intervention using a composite (averaged) score of 13 seven-point scale items (Table 2). In addition, perceptions of the interaction length were assessed with a single seven-point item “How do you feel about the length of the interaction you just had?,” with responses ranging from “Too short” to “Too long.”

**Table 2.** Satisfaction measure items.

Item	Anchor 1	Anchor 7	Mean (SD)
How satisfied are you with the agent?	Not at all	Very satisfied	6.28 (1.18)
How satisfied are you with the instructional experience?	Not at all	Very satisfied	6.50 (0.86)
How much would you like to continue working with the agent?	Not at all	Very much	5.67 (1.67)
How much do you trust the agent?	Not at all	Very much	6.03 (1.25)
How much do you like the agent?	Not at all	Very much	5.80 (1.42)
How easy was interacting with the agent?	Very difficult	Very easy	6.60 (0.62)
How knowledgeable was the agent?	Not at all	Very knowledgeable	6.53 (0.97)
How clear and understandable was the information presented by the agent?	Very unclear	Very clear	6.63 (0.89)
How relevant was the information to your needs and concerns?	Not relevant at all	Extremely relevant	6.03 (1.22)
How likely would you be to recommend this system to other cancer patients?	Very unlikely	Very likely	6.03 (1.35)
How satisfied are you with the role-play interactions?	Not at all	Very satisfied	6.13 (1.38)
How effective were the role-playing interactions?	Not at all	Very effective	5.87 (1.46)
How realistic were the role-playing interactions?	Not at all	Very realistic	5.23 (1.65)
Composite	—^a^	—^a^	6.20 (0.90)

a Not applicable.

### Statistical Analysis

Given the exploratory nature of the study, no formal sample size calculation was conducted. The research data were analyzed using IBM SPSS Statistics (version 28, IBM Corp). The distribution of pre-post differences in KnowGene scores was normal, indicating parametric paired sample t-test use for pre-post tests. All pre-post tests on single scale item Family Communication Measures were tested using non-parametric Wilcoxon Signed Ranks Tests. The distribution of scores for the composite measure of satisfaction as well as intervention duration were non-normal, thus non-parametric tests were used. Descriptive statistics were used to summarize the data. The threshold for statistical significance was set at *P*<.05.

## Results

The 30 participants took an average of 40.87 (SD 19.25, range 18 to 104) minutes to complete the GRACE intervention session. Those who reported having had a genetic test completed spent significantly more time on the intervention (48.1 mins vs 31.4 mins, Mann-Whitney *U*=58.5, *P*=.03).

### Sociodemographic Characteristics of Participants

Table 3 shows the sociodemographic characteristics of the 30 study participants. The age of the participants ranged from 19‐79 years (mean 44.9, SD 16.4 y). A majority of participants identified as male (16/30, 53.3%), were White (24/30, 80.0%), and had at least some college education (25/30, 83.3%). The majority had received prior cancer-related genetic testing (17/30, 56.7%).

**Table 3.** Sociodemographic characteristics of survey participants (N=30).

Characteristics	Values
Age (years), mean (SD)	44.9 (16.4)
Gender, n (%)	
Female	13 (43.3)
Male	16 (53.3)
Transgender	1 (3.3)
Race, n (%)	
White	24 (80.0)
Black	5 (16.7)
American Indian	1 (3.3)
Ethnicity, n (%)	
Hispanic	1 (3.3)
Non-Hispanic	29 (96.7)
Education, n (%)	
High school	5 (16.7)
Some college	8 (26.7)
College graduate	8 (26.7)
Advanced degree	9 (30.0)
Ever had a cancer-related genetic test	
Yes	17 (56.7)
No	13 (43.3)

### Family Communication

Following the interaction with GRACE, participants indicated they were significantly more likely to share genetic test results with their family (Table 1). Participants also indicated that the importance of sharing genetic test results was significantly greater following the intervention, and that they felt significantly more confident in discussing genetic test results with their family. There was only a trending increase in the degree of comfort participants felt in discussing genetic test results with their family, *P*=.07.

### Cancer Genetics Knowledge

KnowGene scores increased from pre-intervention, 13.27 (SD 1.91), to post-intervention, 13.70 (SD .35) but this difference was not significant, paired t_29_=1.25, *P*=.22.

### Usability and Satisfaction

Participants scored GRACE an average of 42.7/60 (71.2%) on the SUS.

Participants rated their satisfaction with GRACE an average of 6.20 (SD 0.90) on the composite measure of satisfaction (Table 2). A single sample Wilcoxon signed ranks test indicated this score (mean 6.2) was significantly greater than a neutral score of 4.0, *P*<.001. There was a significant positive correlation between time to complete the intervention and satisfaction, Spearman ρ=.537, *P*=.002.

## Discussion

### Principal Findings

We aimed to understand usability and the preliminary impact of a digital health intervention to support patients with cancer in communicating genetic testing information to at-risk relatives. Overall, we found that comfort, confidence, and intent to communicate increased, and that participants had high levels of satisfaction with the intervention. Cancer genetics knowledge remained constant. Preliminary results demonstrate that the intervention GRACE should be evaluated further to understand the impact on rates of cascade testing.

### Limitations

Our study had some limitations. First, our sample was a convenience sample of individuals recruited from a research database. We did not select people who had completed cancer genetic testing and who had actual testing information to communicate. We therefore could not measure actual communication or uptake within a family. Additionally, the sample is small and relatively homogeneous, recruited from one online crowdsourcing site; therefore, results may vary as the sample diversifies. Specifically, KnowGene scores were relatively high at baseline, and therefore no change was found. If we targeted patients with varied educational levels and knowledge, we may have noticed an impact. This may have contributed to our lack in change in knowledge scores.

Our study is the first to use a conversational agent to provide a fully virtual and automated intervention to promote family communication between a patient with cancer and at-risk relatives. Other forms of digital health interventions have been tested to improve these outcomes. In a recent meta-analysis focused on interventions to improve cascade genetic testing, only two technology-based interventions were identified [11]. One was the Family Gene Toolkit, a webinar-based intervention to promote communication in families with hereditary breast and ovarian cancer syndrome that also included face-to-face and telephone interactions over 5-weeks [7]. The published data for this study demonstrate acceptability [9], however, outcomes have not yet been published. The other was the Mobile Application for Genetic Information on Cancer (mAGIC) trial which focused on ovarian cancer risk communication using a mobile app over 3-months [6]. Outcomes of this pilot randomized trial demonstrated significant increases in hereditary cancer knowledge and family communication rates for the intervention group compared to control, but no significant differences in use of cancer genetic counseling services.

Three additional trials were identified since the publication of the meta-analysis that use technology to promote cascade testing, mainly through the use of video education, which was found to be non-inferior compared to usual care to provide genetics education [29] and also to not cause distress [30]. The Genetic Education, Risk Assessment, and Testing (GENERATE) Study used telemedicine and online genetics education through videos to reach family members of patients with pancreatic cancer and a known pathogenic variant, finding that it was successful at improving uptake of testing [31]. Two protocol papers were available related to video education trials: the Genetic Education for Men (GEM) trial (focused on men with prostate cancer [32]), and the Facilitated Cascade Testing (FaCT) trial [33].

While providing genetics education information through a video can be useful and effective at providing information, there are limitations that can be addressed through more interactive computer programs such as relational agents. Video education is static and often challenging to keep up to date and current, a need for those seeking genetics information. Additionally, providing information passively without interaction diminishes learning outcomes. Video education is also difficult to tailor for the nuances required within a genetics education setting.

Researchers and companies are now developing chatbots to facilitate testing or family communication both focused on cancer and within other inherited domains, such as familial hypercholesterolemia [34-36] . However, chatbots rely entirely on text-based interaction, limiting their use in low-literacy populations. Accessibility by family members across the literacy spectrum is essential to ensure that all at-risk individuals receive the genetic risk and mitigation information they need, including the 46% of US adults with low or marginal health literacy [37]. Additionally, chatbots driven by large language models, can hallucinate and provide false information [38], even when driven by retrieval augmented generation [39], making them potentially unsafe if their output is not entirely screened by clinicians. Therefore, solutions that provide trustworthy, controlled, and precise information are needed to ensure accurate and appropriate information without intensive provider oversight, and in a manner accessible to all.

### Conclusions

In order to achieve the full public-health impact of cancer genetic testing, it is critical that information about hereditary cancer risk is communicated within families, often originating from the patient with cancer. The GRACE intervention demonstrates an opportunity to motivate patients with cancer to communicate genetic information and promote cancer genetic testing to at-risk relatives. Further large-scale randomized studies are needed to understand the impact of GRACE on genetic testing outcomes.

## Supplementary material

10.2196/87623 Checklist 1 TREND trial checklist.

## Abbreviations

FaCT: Facilitated Cascade Testing
GEM: Genetic Education for Men
GRACE: Genetic Relational Agent for CascadE
mAGIC: Mobile Application for Genetic Information on Cancer
RA: relational agent
SUS: System Usability Scale
TREND: Transparent Reporting of Evaluations with Nonrandomized Designs
